# Type 3 polyglandular autoimmune syndrome: association of lada, hashimoto's thyroiditis and celiac syndrome

**DOI:** 10.1186/1758-5996-7-S1-A91

**Published:** 2015-11-11

**Authors:** Tjia Sian Tjioe, Filipe Torres Lopes, Thais Martinez Salina, Janaina Aparecida Soares Ferro Laranjo, Carmen Elisa Pena Pineda, Alberto K Arbex, Larissa Bianca Paiva Cunha de Sá

**Affiliations:** 1 IPEMED, Mogi das Cruzes, Brazil

## Background

Polyglandular Autoimmune Syndrome (PAS) was first described by Neufeld et al in 1980 as a syndrome involving multiple organs with endocrine disorders with hypofunction of the involved organ. The peak age-specific incidence occurs between 20 to 60 yrs. of age. Type 3 Polyglandular Autoimmune Syndrome is defined as autoimmune thyroiditis in association with Type 1 Diabetes; Type 3A when associated to Sarcoidosis or Celiac Disease, associated to Pernicious Anemia on Type 3B, or with Vitiligo or Alopecia on Type 3C.

## Objective

To report a case of Type 3 Polyglandular Autoimmune Syndrome. Results: SSM, 39 year old male, caucasian, diagnosticated with Type 2 Diabetes 4 yrs. ago was referred to the endocrinology department due to a 10kg weight loss in a 1 month period. He used maximum doses of metformin that was suspended and introduced NPH and Regular insulin improved the glycemic control. He also mentioned a hard-to-control autoimune hypothyroidism in use of high levels of levothyroxine (> 2 mcg/kg of weight) with high levels of Anti-TPO antibodies. On the first medical appointment it was suspected of being an autoimmune diabetes, LADA, confirmed with GAD Antibodies=201,3 and positive islet-cell antibodies. Due to the difficult control of the hypothyroidism, malabsorption was suspected. Therefore starting investigation for Celiac Disease, showing Antitransglutaminase IgA >128, Antigliadin IgA >213 and IgG=17. Upper endoscopy with moderate enanthematic pangastritis and H. pylori infection. Duodenum biopsy with chronic duodenitis with villous atrophy, villus-cript relation 1: 1 and intraepithelial lymphocytes 48 lymphocytes per 100 enterocytes, confirming Celiac syndrome. The patient evolved with abnormal liver enzymes. Abdominal ultrasound and hepatitis serologic tests without alterations raised the hypothesis of an autoimmune hepatitis that was discarded (P-anca, Anti-smooth muscle antibody, Alpha-1-antitrypsin and Ceruloplasmin all non-reagent). Also investigating Addison's disease (normal cortisol). Therefore concluding that the patient suffers from Type 1 Diabetes, Hypothyroidism due to Hashimoto's thyroiditis and Celiac Syndrome, closing the diagnosis of Type 3 Polyglandular Autoimmune Syndrome.

## Conclusion

The hypothesis of PAS should be raised in all patients with autoimmune involvement of organs that make up the syndrome, therefore permitting that an early diagnosis may establish therapies to prevent future complications.

